# Expression of UCOE and HSP27 Molecular Elements to Improve the Stable Protein Production on HEK293 Cells

**DOI:** 10.1155/bmri/5556353

**Published:** 2025-02-25

**Authors:** Concepción Sosa-García, Uriel Abdallah Sánchez-Pacheco, Carlos Alberto Tavira-Montalvan, Angélica Meneses-Acosta

**Affiliations:** Laboratory of Pharmaceutical Biotechnology, Faculty of Pharmacy, Autonomous University of the State of Morelos, Cuernavaca, Morelos, Mexico

**Keywords:** apoptosis, autophagy, HEK293, HSP27, IFN*γ*, programmed cell death, UCOE

## Abstract

Recombinant proteins represent one of the greatest achievements of modern pharmaceutical biotechnology, as they are increasingly used across nearly all branches of medicine to treat a wide range of conditions. In response to this demand, various cell engineering approaches have been developed to improve their expression. Some of these approaches involve the use of genetic elements that prevent the silencing of the gene of interest, as well as the generation of resistant cell lines to inhibit or avoid programmed cell death (PCD). This research focuses on analyzing the effects of overexpression of UCOE elements and the HSP27 protein, both individually and together, on the production of human rIFN*γ* in HEK293 cells. Our results show that 4-Kb UCOE elements have no effect on protein production in HEK293 cells, while overexpression of HSP27 prolongs the stationary phase during growth kinetics. The Qp of rIFN*γ* is 96-fold higher in clones containing the HSP27/UCOE combination compared to the clone containing only UCOE elements or to the control HEK293 cells. These results correlate with the MCP analyses, which showed that overexpression of HSP27 decreased the expression of Bax, caspase 3, cytochrome C, Beclin, and LC3II mRNA. Finally, this study suggests the potential utility of a cell engineering approach based on the overexpression of the human HSP27 protein for enhancing the production of recombinant viruses and proteins in HEK293 cells.

## 1. Introduction

The significant achievement of recombinant therapeutic proteins (RTPs) in modern medicine underscores the need for continuous improvement in their production processes to meet growing therapeutic demands. The demand for RTPs has surged, with the FDA and EU approving 178 products from 2015 to 2019 and 117 products from January 2020 to June 2022 [1]. This growth is largely attributed to mammalian cell–based production platforms, which provide essential posttranslational modifications, such as glycosylation, that influence the quality, efficacy, and safety of these biopharmaceuticals [2]. To address this increasing demand, innovative strategies are being explored to enhance expression levels in animal cell cultures, previously seen as limited for production. Key goals include boosting cell viability, viable cell density, and cell-specific production rates to ensure a reliable supply of high-quality RTPs [3].

Epigenetic silencing of genes significantly contributes to the loss of productivity in recombinant protein production. This silencing occurs through modifications such as hypermethylation of CpG sequences and histone hypoacetylation [4, 5]. To address this, molecular strategies involving elements that counteract these effects are essential. Novel expression vectors incorporating genetic elements, such as insulators, have been developed to prevent the spread of repressive chromatin [6], including UCOE vectors. UCOE elements are nucleotide sequences that provide stable, integration-independent transgene expression, even in heterochromatin regions like centromeres [7]. These elements consist of two promoters from the housekeeping genes HNRPA2B1 and CBX3, surrounded by methylation-free CpG islands and euchromatic histone marks, which promote stable gene expression.

The incorporation of UCOE elements into expression vectors has been shown to significantly improve recombinant protein production in some cases [8–10]. While such approaches have primarily been explored in animal cell systems such as CHO cells, there is increasing interest in applying them to other platforms, such as HEK293 cells. This may offer significant advantages to the biotechnology industry, highlighting the potential of UCOE elements to improve transgene expression in various production systems.

On the other hand, molecular strategies targeting the modulation of genes related to programmed cell death (PCD) are employed to enhance cell development and improve bioprocess outcomes [11]. PCD significantly impacts bioprocesses by reducing cell viability and product quality. To address this, genes involved in PCD regulation are either inserted or deleted to extend the lifespan of cell cultures. This includes downregulating or deleting proapoptotic genes such as Bax, Bak, caspase 3, and caspase 7 [12–14] and overexpressing antiapoptotic genes from the B cell lymphoma protein family, such as Bcl-xL and Bcl-2 [15–17]. Additionally, heat shock proteins such as HSP27 are utilized in these strategies to further support cell survival and productivity. HSP27 is a crucial chaperone involved in cytoskeletal stability, protein synthesis, redox potential, and apoptosis modulation [18]. Recent studies suggest its involvement in autophagy, although it is primarily studied as a biomarker for cancer and neurodegenerative diseases [19, 20]. Molecular applications have shown that overexpression of HSP27 in murine systems modulates apoptosis pathways and delays caspase activity, enhancing the specific productivity of target proteins [21, 22].

Finally, HEK293 cells are used industrially for producing recombinant proteins, virus-like particles (VLPs), and viral vectors for biopharmaceuticals, vaccines, and gene therapy products [23, 24]. This cell line was originally derived from human embryonic kidney cells and subsequently transfected with the E1 region of Adenovirus Type 5, which was integrated into Chromosome 19 [25, 26]. HEK293 cells, being of human origin, can perform posttranslational modifications, such as glycosylation, that are similar to those in humans [27], which is a major advantage. Additionally, these cells can be adapted to grow in both suspension and adherent cultures [28]. However, some challenges remain, such as their productivity compared to CHO cells.

Based on the above, this study focuses on analyzing the effect of UCOE elements and HSP27 protein expression, both individually and together, as potential molecular tools for the development of stable HEK293 cell clones, with a direct impact on recombinant proteins production.

## 2. Materials and Methods

To improve the productivity of biopharmaceuticals using HEK2923 cells, various molecular strategies have been employed. In this context, UCOE elements and the HSP27 protein have shown promise as molecular targets for enhancing productivity in CHO cells. However, there is limited scientific evidence analyzing the productivity of these elements individually, and no evidence exists regarding their combined effect within the HEK293 production system. This underscores the importance of conducting such an analysis, as presented in our study. For this purpose, different stable expression clones were generated: HEK293/UCOE (HEK/UCOE) clone and HEK293/HSP27 (HEK/27) clone for the analysis of each strategy separately and the HEK293/HSP27/UCOE (HEK/27/U) clone for the synergistic evaluation. To obtain these clones, two expression vectors were constructed.

### 2.1. Genetic Vector Construction

Two expression vectors were created: the UCOE/rIFN*γ* vector using the UCOE-Human 4Kb Puro Set expression vector from Merck and the TOPO/HSP27 vector using the pcDNA3.3-TOPO plasmid. The human HSP27 gene (GenBank Accession No. NM_001540.5) was obtained from the Frt-hspB1 vector. For the human IFN*γ* gene (GenBank Accession No. NM_000619.3), the pVAX1/IFN*γ* vector was used, along with the oligos listed in Table 1. The human IFN*γ* gene encodes for the model transgenic IFN*γ* protein, a dimerized soluble cytokine weighing between 15.5 and 25 kDa. The protein possesses native N-glycosylation at residues N25 and N97, as well as an N-terminal pyroglutamate residue.

#### 2.1.1. Vector Construction and Characterization

The transgene inclusions (IFN*γ* and HSP27) required to generate the respective vectors were performed using PCR reactions with the oligos listed in Table 1. The integrity of the gene sequences was confirmed at the DNA Synthesis and Sequencing Unit (USSDNA) of the IBT-UNAM. The obtained sequences were analyzed by Chromas 2.6.6 (https://technelysium.com.au/wp/chromas/) and Blast from NCBI (https://blast.ncbi.nlm.nih.gov/blast/Blast.cgi).

### 2.2. Cell Cultures

Adherent cultures of human embryonic kidney cells (HEK293, ATCC CRL-1573) were maintained in D-MEM/F-12 medium (Gibco) supplemented with 10% (vol/vol) fetal bovine serum (FBS). The cultures were incubated at 37°C and 5% CO_2_. Cell Passage Number 8 was used for the experiments to ensure a low passage number and minimize the risk of genetic modifications.

#### 2.2.1. Clone Generation for Stable Expression

Three stable clones were constructed: HEK293/UCOE, HEK293/HSP27, and HEK293/HSP27/UCOE. The HEK293/UCOE and HEK293/HSP27 clones were generated via lipofection using Lipofectamine 2000 (Lipofectamine 2000, Thermo Fisher Scientific) following the manufacturer's instructions. The HEK293/HSP27 clone was subsequently retransfected with the UCOE/rIFN*γ* vector to generate the HEK293/HSP27/UCOE clone. Prior to transfection, the vectors were linearized using specific restriction enzymes. Monoclonal selection was performed using the limiting dilution technique. The selection medium consisted of DMEM/F12 (10% SFB) with Puromycin (Sigma, Ref. P9620) was used for selecting UCOE/rIFN*γ* clones, while Geneticin (Gibco, Ref. 10131–027) was used for selecting TOPO/HSP27 clones. A stable clone was defined as one maintaining viability for more than 16 generations under selective pressure.

#### 2.2.2. Clonal Evaluation

To confirm the successful generation of each clone, the presence of rIFN*γ* and HSP27 messenger RNA was verified using RT-PCR for the corresponding clones. Total RNA was extracted using TRIZOL (TRI Reagent, Zymo Research Cat. No. R2050-1–50) following the manufacturer's instructions. Genomic DNA was removed using the DNaseI enzyme (Thermo Scientific, Cat. No. EN0521). Complementary DNA (cDNA) synthesis was performed with RevertAid H Minus First Strand cDNA Synthesis kit (Thermo Scientific, Cat. No. K1631). The oligonucleotides used to amplify IFN*γ* and HSP27 cDNA are listed in Table 1.

rIFN*γ* by the HEK293/UCOE and HEK293/HSP27/UCOE selected clones was quantified using ELISA with the Human IFN gamma ELISA Kit (Invitrogen, BMS228), following the manufacturer's instructions.

#### 2.2.3. Effect of UCOE and HSP27 Elements on the Production of Recombinant Proteins

To evaluate the effect of the individual or combined expression of UCOE and HSP27 elements on the production of rIFN*γ*, the growth kinetics of each obtained clone were analyzed. Cell viability and kinetic and metabolic parameters were determined for each clone. rIFN*γ* was quantified using the ELISA method to calculate volumetric production and specific productivity (Qp). A detailed description is provided below.

##### 2.2.3.1. Kinetic and Metabolic Profiling of HEK293/UCOE, HEK293/HSP27, and HEK293/HSP27/UCOE Clones

Production kinetics were evaluated in 12-well plates, where 5 × 10^4^ cells/mL of each generated clone were seeded and cultured in static conditions using DMEM/F-12 medium supplemented with 10% of FBS. The cultures were incubated at 37°C and 5% CO_2_ for 12–16 days. Cell pellets were collected every 24 h for cell counting using the trypan blue exclusion method. Culture supernatants were analyzed to quantify the production of human rIFN*γ* using the Invitrogen Human IFN gamma ELISA Kit (BMS228), following the manufacturer's instructions. Additionally, cell metabolism was assessed by measuring monitored by quantification of glucose, glutamine, lactate, and glutamate levels using the YSI model 2900 analyzer. From the collected data, the volumetric production, Qp, and the kinetic and metabolic parameters of each clone were determined. The results are expressed in relative units, with the HEK293/UCOE clone used as a control, to compare the production levels of human rIFN*γ* the different clones.

Qp was calculated as a function of growth rate (*μ*) and volumetric productivity using the following equation:

 $$\begin{array}{c} \text{Qp}=\mu *\frac{P2-P1}{N2-N1} \end{array}$$

where *P* represents the volumetric productivity and *N* is the viable cell density.

##### 2.2.3.2. Evaluation of the Effect of HSP27 as a Modulator of PCD

To assess the impact of HSP27 on PCD, changes in the expression of apoptosis and autophagy-related genes were analyzed using RT-PCR. Additionally, apoptosis and autophagy fluxes were evaluated using dyes such as acridine orange (AO), propidium iodide (PI), and a green fluorescent detection reagent, observed via epifluorescence microscopy. RT-PCR: RNA extraction and conversion to cDNA were performed using standard methods. Specific primers for apoptosis and autophagy-related genes are listed in Table 1.

AO and PI (AO/PI) staining: HEK293 and HEK293/HSP27 cells were cultured on round coverslips in 24-well plates and treated with 0.195 nM staurosporine (STS) to induce apoptosis. The cells were stained with a 1:1 mixture of AO and PI and observed under a Nikon eclipse E400 epifluorescence microscope using TRITC (red) and FITC (green) filters.

Autophagosome staining: HEK293 and HEK293/HSP27 cells were cultured to 90% confluence, washed with PBS, and incubated with fresh medium with or without serum for 48 h. Cells were then treated with the Autophagy Detection Kit (Abcam, ab139484) and observed under a Nikon eclipse E400 epifluorescence microscope, following the manufacturer's protocol.

### 2.3. Statistics

Results from densitometry and growth kinetics analyses were expressed as the mean ± standard deviation of six samples. Yields were expressed as the mean ± standard error of two samples, while, for volumetric production, a two-way ANOVA (*p* < 0.05) was performed, followed by Tukey's post hoc test. For specific productivity, a one-way ANOVA (*p* < 0.05) was conducted, also followed by Tukey's post hoc test. In both cases, results were expressed as the mean ± standard error of two samples. RT-PCR results were presented as the mean ± standard deviation, and statistical significance was determined using the Mann–Whitney test (*p* < 0.05). Graphs and statistical analysis were performed in GraphPad Prism 9.0 with a significance value < 0.05.

## 3. Results and Discussion

### 3.1. Construction and Characterization of UCOE/IFN*γ* and TOPO/HSP27 Vectors

**Figure 1:**
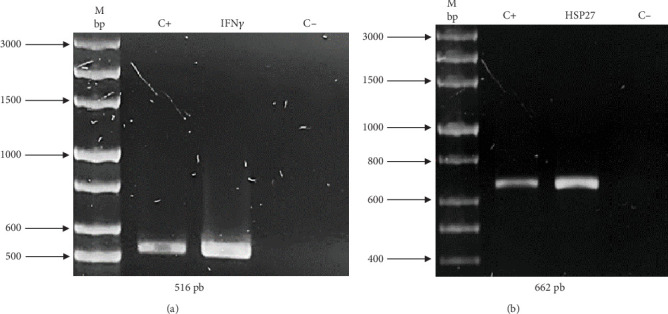
TOPO/HSP27 and UCOE/IFN*γ* vector constructs. In order to confirm the presence of the HSP27 and human IFN*γ* genes in the corresponding construct, PCR and sequencing of each vector were performed. (a) PCR of UCOE/IFN*γ* vector, C+: pVAX1/IFN*γ* vector, IFN*γ*: UCOE/IFN*γ* vector (516 bp). (b) PCR of the TOPO/HSP27 vector, C+: Frt-HSPB1 vector, HSP27: TOPO/HSP27 vector (662 bp). M bp: base pair marker, C−: negative control of the reaction.

As previously described, two expression vectors were constructed to demonstrate the effectivity of UCOE and HSP27 protein. The presence of genes of interest (human rIFN*γ* and HSP27) within the constructs was validated by PCR.  Figure 1(a) shows an amplicon of 516 bp corresponding to IFN*γ*, and Figure 1(b) shows an amplicon of 662 bp corresponding to HSP27. The sequence integrity of the IFN*γ* and HSP27 genes within the vectors was confirmed by sequencing (Supporting Information). Figure S1 shows the sequencing results for the UCOE/IFN*γ* plasmid, showing 100% identity of the IFN*γ* sequence in the UCOE vector with the coding region registered in the NCBI database. Similarly, Figure S2 displays the sequencing results for the TOPO/HSP27 vector, also showing 100% identity of the HSP27 sequence in the TOPO vector with the NCBI database. In both cases, the gene sequence was confirmed to be in the correct reading frame.

Once the presence and integrity of the genes were confirmed, these vectors were used to stably transform HEK293 cells.

### 3.2. Clonal Evaluation and Selection

#### 3.2.1. HEK293/UCOE

**Figure 2:**
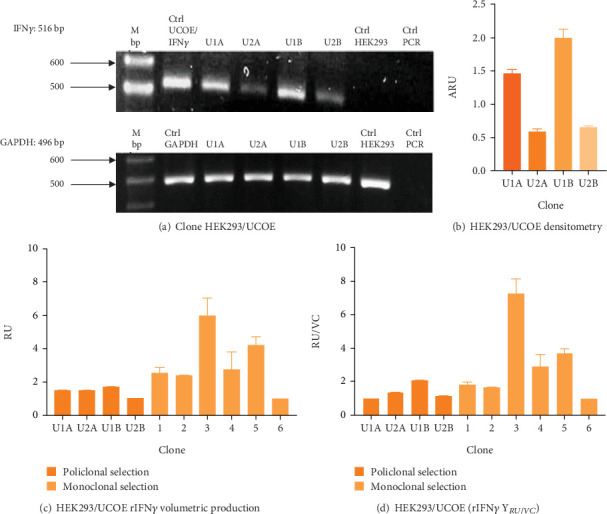
HEK293/UCOE clone evaluation. (a) rIFN*γ* RT-PCR. U1A: Clone 1A, U2A: Clone 2A, U1B: Clone 1B, U2B: Clone 2B, Ctrl UCOE/rIFN*γ*: UCOE/rIFN*γ* construct. (b) Densitometric analysis of HEK293/UCOE clone. Increased expression of human rIFN*γ* messengers was observed in the U1B clone. Results are expressed as mean ± standard deviation of three samples. (c) Volumetric production of human rIFN*γ* during polyclonal and monoclonal selection. Results are expressed as the mean ± standard error of two samples. (d) Human rIFN*γ* yields during polyclonal and monoclonal selection. Results are expressed as mean ± standard error of two samples. During polyclonal selection, a higher production of human rIFN*γ* was observed in Clone U1B, which was used for monoclonal selection, where Clone 3 was identified as the one with the highest production and was used for subsequent analysis.

Figure 2(a) shows the RT-PCR results of the polyclonal pools, with an amplicon of 516 bp, confirming the presence of the transgene in the four generated pools (U1A, U2A, U1B, U2B). In this case, the presence of the rIFN*γ* gene is not observed in the parental cell line, as it is not produced endogenously, and the parental cells were used as a negative control. The mRNA expression level of rIFN*γ* (Figure 2(b)) was the highest in pool U1B (1.99 ± 0.14 ARU). Additionally, Figures 2(c) and 2(d) show that this pool exhibited high volumetric production and yields of rIFN*γ* (1.73 ± 0.03 RU and 2.05 ± 0.02 RU/VC, respectively). Based on these results, monoclonal selection was performed from this pool.

To analyze the effect of UCOE elements, the six highest yielding clones derived from monoclonal selection were examined. Figures 2(c) and 2(d) show that the clone with the highest titer and yields was Clone 3 (5.96 ± 0.75 RU and 7.24 ± 0.91 RU/VC, respectively), which exhibited a sevenfold higher yield compared to the lowest-producing clone.

#### 3.2.2. HEK293/HSP7/UCOE

**Figure 3:**
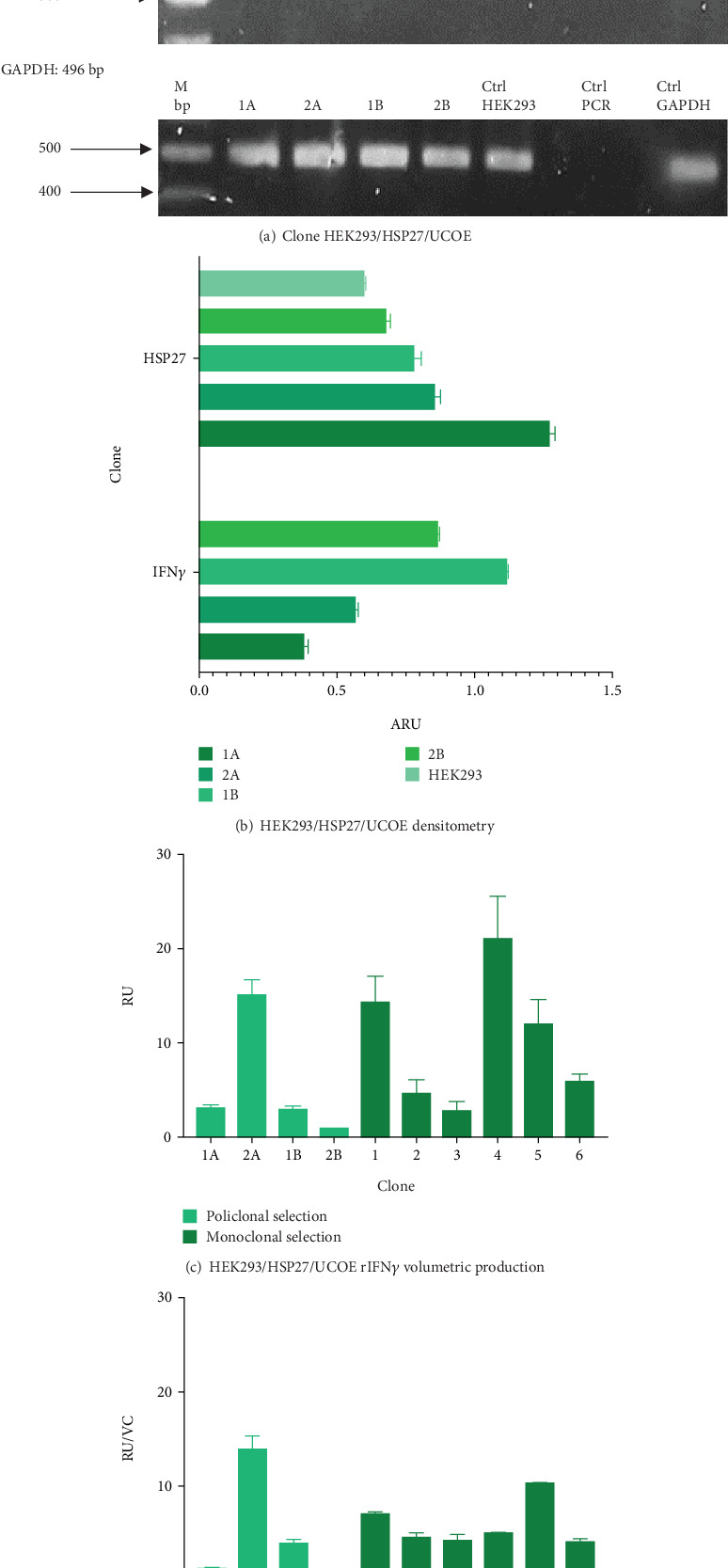
HEK293/27/UCOE clone evaluation. (a) rIFN*γ* and HSP27 RT-PCR. 1A: Clone 1A, 2A: Clone 2A, 1B: Clone 1B, 2B: Clone 2B, Ctrl UCOE/IFN*γ*: UCOE/IFN*γ* construct, Ctrl PCR: control of PCR reaction, HSP27 RT-PCR. CA: Clone A, CB: Clone B, Ctrl TOPO/HSP27: TOPO/HSP27 construct, Ctrl HEK293: nontransfected HEK293, Ctrl GAPDH: GAPDH constitutive gene. (b) Densitometric analysis of HEK293/27/UCOE clone. Increased expression of human rIFN*γ* messengers is observed in the 1B clone; in the case of HSP27 messengers, higher overexpression is observed in Clone 1A. Results are expressed as mean ± standard deviation of three samples. (c) Volumetric production of human rIFN*γ* during polyclonal and monoclonal selection. Results are expressed as mean ± standard error of two samples. (d) Human rIFN*γ* yields during polyclonal and monoclonal selection. Results are expressed as mean ± standard error of two samples. During polyclonal selection, a higher production of human rIFN*γ* was observed in Clone 2A, which was used for monoclonal selection, and the three clones with the highest production (1, 4, and 5) were chosen for further analysis.

To evaluate the effect of the HSP27 protein in combination with the UCOE elements, four polyclonal pools (1A, 2A, 1B, and 2B) were generated.  Figure 3(a) confirms the presence of the HSP27 gene, with an amplicon of 142 bp in all cases. According to the densitometric analysis (Figure 3(b)), Pool 1A shows overexpression of this protein compared to the parental line, likely due to the effect of the TOPO/HSP27 plasmid. The expression level in Pool 1A is 2.11 times higher than in the parental clone, with the HSP27 mRNA expression level at 1.27 ± 0.02 ARU. Meanwhile, the mRNA expression level of rIFN*γ* was highest in Pool 1B (1.12 ± 0.003 ARU).

Thus, Pool 3A was used for this purpose, with a titer of 15.25 ± 1.50 RU and a yield of 13.94 ± 1.37 RU/CV (see Figures 3(c) and 3(d)). Next, the six highest-yielding clones were selected (Figures 3(c) and 3(d)). When comparing the average titer and yield in both populations, a higher expression of the protein of interest was observed in the presence of both molecular markers (3.2- and 1.9-fold, respectively).

To further analyze the effect of these tools, three clones with the highest titer and yields were selected: Clone 1 (14.34 ± 2.83 RU and 7.00 ± 0.19 RU/VC), Clone 4 (21.18 ± 4.51 RU and 4.96 ± 0.050 RU/VC), and Clone 5 (11.98 ± 2.69 RU and 10.36 ± 0.02 RU/VC). These clones were designated as HEK/27/U-C1, HEK/27/U-C4, and HEK/27/U-C5.

#### 3.2.3. HEK293/HSP27

**Figure 4:**
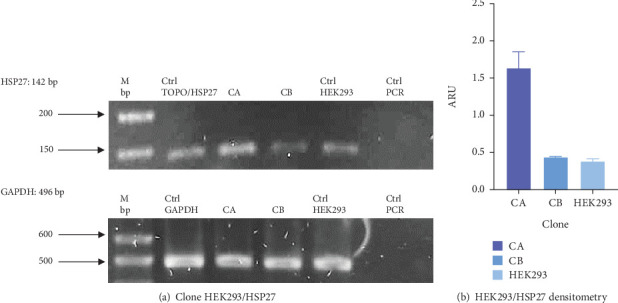
HEK293/HSP27 clonal evaluation. The presence of the gene human HSP27 of each clone was performed by RT-PCR. (a) HSP27 RT-PCR. CA: Clone A, CB: Clone B, Ctrl TOPO/HSP27: TOPO/HSP27 construct, Ctrl HEK293: nontransfected HEK293, Ctrl GAPDH: GAPDH constitutive gene. (b) Densitometric analysis of HEK293/HSP27 clone. Increased expression of HSP27 messengers was observed in the CA clone. Results are expressed as mean ± standard deviation of three samples.

Figure 4(a) shows the presence of HSP27 protein transcripts in the two clones derived from the monoclonal selection, with an amplicon of 142 bp observed in the CA and CB clones, as well as in the HEK293 Ctrl Lane (nontransfected cells). To verify overexpression due to the effect of TOPO/HSP27 vector transfection, densitometric analysis was performed (Figure 4(b)). The result shows that the level of HSP27 mRNA expression was higher in Clone CA (1.64 ± 0.22 ARU) compared to Clone CB (0.44 ± 0.009 ARU). The increase in HSP27 expression in Clones CA and CB was 4.1- and 1.1-fold higher, respectively, compared to the parental clone. Therefore, the CA clone was chosen for further analysis to generate the HEK293/HSP27/UCOE clone.

### 3.3. Effect of UCOE Elements and Overexpression of HSP27 Protein on the Growth and Biochemical Profile of the Clones Generated

**Figure 5:**
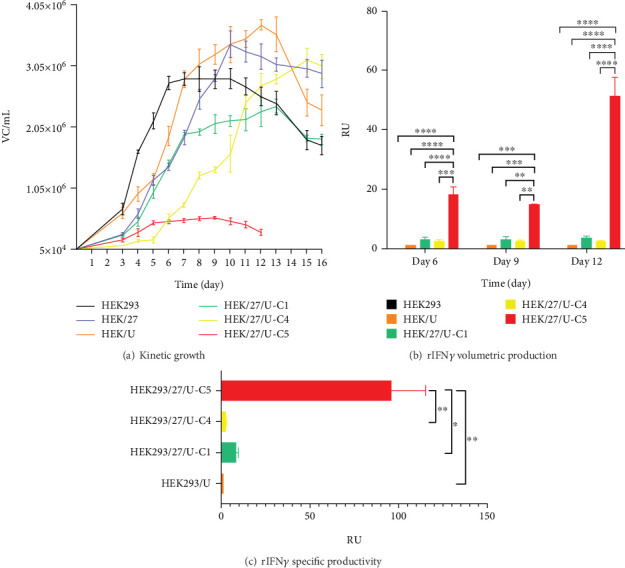
Growth kinetics of the HEK293 clones generated and volumetric production and specific productivity of human rIFN*γ*. (a) Growth kinetics were carried out in static cultures with DMEMF12 medium (10% FBS). Supernatant collection and cell counts were performed every 24 h. Results are expressed as mean ± standard deviation of six samples. (b) From the supernatants collected from Days 6, 9, and 12 of the growth kinetics/production kinetics of human rIFN*γ*, the volumetric production of the rIFN*γ* producing clones was evaluated. A two-way ANOVA (*p* < 0.05) and a Tukey analysis were performed as a post hoc test. (c) The specific productivity of human rIFN*γ* was evaluated from the supernatants collected from Days 6, 9, and 12 of the growth kinetics/production kinetics of human rIFN*γ* and the CV/mL presented at those times; the results show a significant difference between the production of clone HEK293/27/U-C5 and the rest of the clones. A one-way ANOVA (*p* < 0.05) and a Tukey analysis were performed as a post hoc test. The level of significance is taken at 0.05 or 5%. In both cases, results were expressed as the mean ± standard error of two samples. HEK293/HSP27 (HEK/27), HEK293/UCOE (HEK/U), HEK293/HSP27/UCOE-C1 (HEK/27/U-C1), HEK293/HSP27/UCOE-C4 (HEK/27/U-C4), HEK293/HSP27/UCOE-C5 (HEK/27/U-C5).

Figure 5(a) shows the different growth patterns exhibited by the evaluated clones, and Table 2 summarizes the kinetic parameters. The growth rate (*μ*) was determined to be in the range of 0.0146–0.0233 h^−1^, which is consistent with the values reported by [36] (0.014–0.023 h^−1^) and [37] (0.020–0.029 h^−1^) [36, 37] for this production platform. When comparing the three different clones, an increase in doubling time was observed relative to the parental line (HEK293).

In terms of maximum cell density achieved per batch, HEK/27 and HEK/27/U-C4 clones showed only a 1.2- and 1.1-fold increase over the parental HEK293 clone, which was not statistically significant (Table 2). Studies by Amini et al. overexpressed HSP27 in CHO cells to evaluate its effect on cell viability and growth. They observed a threefold increase in the peak viable cell density of clones transfected with HSP27 [38]. Therefore, the results obtained in the present study indicate a lower effect of HSP27 in human-derived cells compared to that reported with the murine platform. In fact, the HEK/27/U-C5 clone only reached a maximum density of 5.72 × 10^5^ cell/mL in a small-scale culture.

On the other hand, the analysis of the metabolic profile was performed by determining the specific consumption and production rates of metabolites; lactate production rates from glucose were also calculated (Table 2). It can be observed that two of the rIFN*γ*-producing clones, HEK/27/U-C1 and HEK/27/U-C5, have the highest lactate production rates.

### 3.4. Effect of UCOE Elements and Overexpression of HSP27 Protein on the Production of Human rIFN*γ*

Regarding volumetric production, it was observed that the clones reached their highest concentration on the 12th day, including HEK/UCOE (control), HEK/27/U-C1, HEK/27/U-C4, and HEK/27/U-C5 (Figure 5(b)). Statistical analysis revealed that HEK/27/U-C5 clone exhibited a significant increase in volumetric production (51.25 ± 6.42 RU) compared to the rest of the clones. This clone also showed a similar effect in terms of Qp (Figure 5(c)), with a value of 96.05 ± 19.21 RU, which was 11.3 times higher with respect to the second most productive clone.

By correlating the metabolic profile and kinetic behavior, it can be concluded that the HEK/27/U-C5 clone demonstrates limited cell growth, focusing its metabolism on recombinant protein production rather than cell division.

#### 3.4.1. Effect of UCOE Elements

When comparing the clone containing only the UCOE elements (HEK/UCOE) to those containing the HSP27 gene, the results show no improvement in volumetric production or Qp of IFN*γ* (Figures 5(b) and 5(c)).

Several researchers have used CHO cells combined with UCOE elements for the stable production of recombinant proteins, achieving high expression levels [8, 39–41]. However, low levels of recombinant protein expression have also been reported in the CHO/UCOE cell combination compared to the use of other chromatin-modulating elements, such as STARs, MARs, and cHS4 [42].

In the case of HEK293 cells, there is limited evidence regarding the use of UCOE elements. However, Bandaranayake et al. reported an increase in the protein of interest [43], which contrast with the findings of [44], who observed lower expression levels compared to control cells or those produced by other chromatin-modulating elements [44]. The difference between the two experiments may lie in the molecular size of the UCOE elements used.

By compiling the data reported so far across various cell lines, the differences in the production levels of the model protein could be attributed to the size of the subfragment of the UCOE element used.  Table 3 presents an analysis supporting this hypothesis. As observed, shorter fragments are more effective in preventing silencing and confer higher expression of the protein of interest, provided that this region belongs to the CBX3 locus [47]. However, this is not a universal rule, and further data are needed to confirm this conclusion.

Therefore, in the present work, using a 4-Kb UCOE element, with a portion of the promoters divergently transcribed from HNRPA2B1 and CBX3, helps explain the absence of a positive effect on the increase in production. This result complements the data in Table 3 for the HEK293 platform. Further studies using fragments smaller than 1.5 Kb are recommended to confirm this trend across other production systems.

#### 3.4.2. Effect of HSP27 Protein Overexpression

The results show that clones overexpressing human HSP27 exhibit higher IFN*γ* production. The average of the six clones with the PCD-modulating protein shows a 3.22-fold higher titer compared to the average of the clones containing only the UCOE elements. In terms of yield, this increase is 1.92-fold. When analyzing the specific productivity of the highest-yielding clones from each population (HEK/U vs. HEK/27/U-C5), it was found to be 96-fold higher.

This behavior could be attributed to the antiapoptotic and chaperone characteristics of HSP27, which is consistent with the findings reported by [21, 22]. Their results demonstrate that overexpression of murine HSP27 in CHO cells enhances apoptosis resistance and increases cell production. Both studies reported enhanced model protein expression as well as delayed activity of caspases 2, 3, 8, and 9 [21, 22].

When analyzing the growth kinetics of the clones overexpressing HSP27 (Figure 5(a)), it was observed that a relatively constant viable cell density was maintained during the stationary phase, compared to the parental cells and the HEK/U clone, which showed a constant decline in viable cell density starting from Day 10 to 12, respectively. This effect could positively impact the batch culture lifetime, potentially increasing the volumetric production of the protein of interest.

It is worth mentioning that, in all cases, approximately 85% viability was obtained at the end of culture (Table 2). However, this was relative to the viable cell density at the end of the culture. This effect may be attributed to the autophagic characteristic of HEK293 cells [49]. To further analyze the activity of HSP27 on PCD, the following studies were performed.

### 3.5. Overexpression of HSP27 Protein Exerts an Effect on the Modulation of Apoptosis and Autophagy

#### 3.5.1. Apoptosis Assays

**Figure 6:**
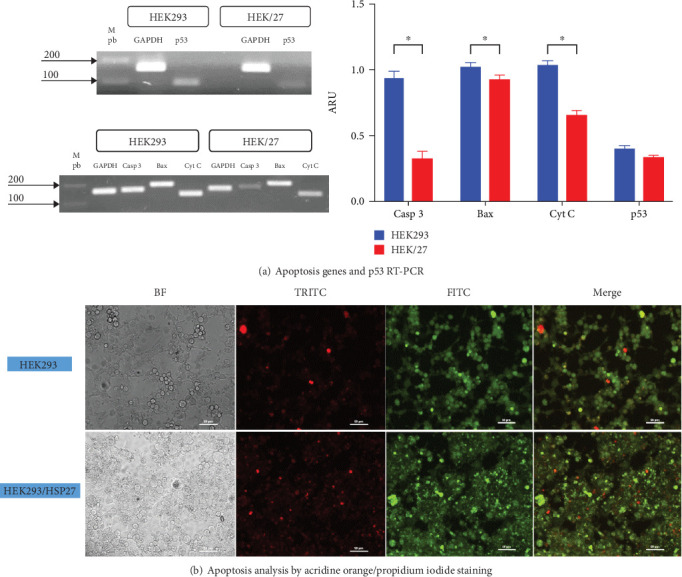
Apoptosis detection. (a) RT-PCR of caspase 3, Bax, cytochrome C, and p53 genes shows significant differences in the expression of these genes between HEK and HEK293/HSP27 (HEK/27) cells, except for p53. Results represent mean ± standard deviation of three samples, and a Mann–Whitney analysis was performed (*p* < 0.05). The level of significance is taken at 0.05 or 5%. (b) Apoptosis analysis was performed by staining cells with acridine orange/propidium iodide. Cells were observed under a Nikon eclipse E400 epifluorescence microscope with 20 × objective.

A possible modulation of apoptosis was analyzed at the transcriptional level by RT-PCR, monitoring the gene expression of caspase 3, Bax, cytochrome C, and p53 (Figure 6(a)). The result demonstrated a significant downregulation of messenger RNA expression in the HEK/27 clone compared to the parental HEK293 clone, except for p53.

DNA damage activates p53 and its downstream target genes, which can lead to either apoptosis or cell survival, through mechanisms such as cell cycle arrest or DNA repair. The roles of HSP27 in p53-mediated cellular responses to DNA damage remain controversial [50]. Some studies suggest that HSP27, depending on its phosphorylation status, plays different roles in regulating the p53 pathway and cell survival. Others research indicates that negative regulation of HSP27 is associated with activation of the p53 pathway [50–52].

Numerous studies support the obtained data, as it is known that HSP27 positively regulates the PI3-activating kinase Akt, which interacts with Bax (Akt/Bax). This interaction blocks the translocation of Bax to mitochondria, preventing the release of cytochrome C [53]. Additionally, HSP27 interacts with cytochrome C and caspase 3, inactivating them [54]. These molecular mechanisms enable HSP27 to modulate apoptosis, which explains the observed decrease in its expression. Furthermore, it has been reported that this function promotes greater cell survival, correlating with the findings in its kinetic profile. However, the same effect was expected for p53, as HSP27 is known to inactivate this gene [55]. Although the results show a decrease in p53 expression, these changes are not statistically significant.

The effect was also monitored by epifluorescence microscopy.  Figure 6(b) shows the results of AO and PI staining of HEK293 and HEK/27 cells treated with STS. STS is known to be one of the most potent and widely used proapoptotic stimuli, as it causes inactivation of factors such as eIF4F and eIF2*α*, as well as protein kinases like p70S6, which are critical for mRNA translation control [56]. STS can induce apoptosis through both the mitochondrial and endoplasmic reticulum stress pathway. Studies have shown that cells with overexpressing apoptosis inhibitors, such as Bcl-2 and Bcl-x(L), when treated with STS, only attenuate but do not completely prevent STS-induced apoptosis [57].

As shown, red nuclei were observed, indicating compromised cell membrane integrity. Morphological features consistent with a PCD process were also identified, including DNA condensation, the presence of intracellular vacuolization, loss of adhesion, nuclear fragmentation, and loss of cytoplasmic structure [58].

#### 3.5.2. Autophagy Detection

To determine whether HSP27 overexpression affects autophagic flux, RT-PCR analysis of Beclin 1 and LC3II was performed, along with fluorescence microscopy in HEK293 and HEK/27 cells. In the autophagy process, proteins such as Beclin 1 and LC3II play a key role, as they are essential for the autophagosome formation. For this reason, they are widely accepted as key markers of autophagic activity [59].

**Figure 7:**
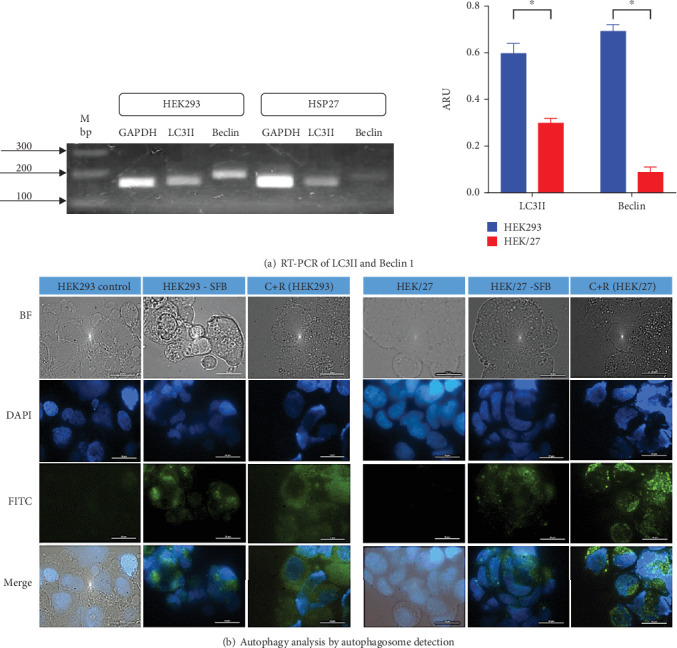
Autophagy detection. (a) RT-PCR of LC3II and Beclin 1 genes shows significant differences in the expression between HEK293 and HEK293/HSP27 (HEK/27) cells. Results represent mean ± standard deviation of three samples, and a Mann–Whitney analysis was performed (*p* < 0.05). The level of significance is taken at 0.05 or 5%. (b) Autophagy analysis was performed by autophagosome detection using the Autophagy Detection Kit (Abcam, ab139484). Nuclei are shown in blue (Hoechst dye, DAPI filter) and autophagic vacuoles (autophagosomes) are shown in green (FITC filter). Cells were observed under a Nikon eclipse E400 epifluorescence microscope with 100 × objective.

RT-PCR analysis for Beclin 1 and LC3II (Figure 7(a)) reveals that mRNA expression of both genes is significantly lower in clone HEK/27 compared to clone HEK293. Although Beclin 1 levels are reduced, it is notable that LC3II levels do not decrease drastically. This observation aligns with studies conducted by Quiles et al. [60], which indicated that autophagosome formation in response to stress can occur independently of Beclin 1 [60].

Figure 7(b) shows the fluorescence microphotographs, where nuclei appear in blue (Hoechst dye, DAPI filter), and autophagic vacuoles (autophagosomes) are shown in green (FITC filter). Rapamycin was used a positive control due to its strong autophagy-inducing properties, while chloroquine served as an inhibitor of autophagosomes degradation to allow their visualization [61, 62]. As expected, in the positive controls for both cell line, autophagosomes are distributed throughout the perinuclear region and cytoplasm. However, upon exposing HEK293 and HEK/27 cells to a mild inducer (FBS deprivation), autophagosomes were predominantly concentrated in the perinuclear region. When comparing the fluorescence intensity and therefore the abundance of autophagosomes, it was notably lower in HEK/27 cells. This observation suggests that HSP27 may protect the cells from undergoing autophagy under the experimental conditions analyzed.

These results suggest that HSP27 downregulates both Beclin 1 and LC3II and exhibits an apparent decrease in autophagosome formation when exposed to a nutrient-deficient environment, such as FBS deprivation. Although this type of PCD is uncommon during bioprocessing, HEK293 cells have the capacity to activate this pathway in the final stage of a batch culture. Therefore, HSP27 appears to exert a modulatory effect on autophagy, leading to metabolic change that enhances the production of recombinant protein.

## 4. Conclusion

In the present study, the effects of UCOE elements and overexpression of human HSP27 protein on the production of human IFN*γ* produced in batch cultures of HEK293 cells were evaluated. The results demonstrated that the generated clones (HEK293/HSP27, HEK293/UCOE, and HEK293/HSP27/UCOE) exhibit different growth patterns compared to the parental HEK293 clone.

The HEK293/UCOE clone showed the lowest volumetric production, suggesting that the size of UCOE element (4 Kb) plays a critical role and may not be ideal for the HEK293 system in this context. In contrast, clones overexpressing HSP27 protein exhibit significantly higher volumetric production (51.24 ± 9.08 UR at 12 days) and Qp (96.05 ± 27.17 UR) indicating a direct relationship between the overexpression of this PCD modulator protein and increased recombinant protein production. These findings correlate with a decrease in Bax, caspase 3, and cytochrome C transcripts in the apoptosis study, as well as a reduction in Beclin 1 and LC3II levels in the autophagy analysis. This study highlights the utility of a molecular strategy based on the overexpression of human HSP27 protein to develop HEK293 cell lines with enhanced recombinant protein production capabilities.

## Figures and Tables

**Table 1:** Designed oligos for RT-PCR reactions.

**Gene**	**Amplicon (bp)**	**Sequence**	**Reference**
*Oligos used for vector construction and clonal evaluation*			
Human IFN*γ*	516	Fwd: 5⁣′-CGGCGGCCGGCCGATGAAATATACAAGTTATATC-3⁣′ Rev: 5⁣′-CTTGTTAGCTAGCTTACTGGGATGCTCTTCGAC-3⁣′	NA
Human HSP27	662	Fwd: 5⁣′-CTTTAATAGGCCGGCCTCGAGAGCATGACCGAGCG-3⁣′ Rev: 5⁣′-CTTAATTAGGCCGGCCGCGTTTACTTGGCGGCAG-3⁣′	NA
RT-PCR HSP27	142	Fwd: 5⁣′-GTCCCTGGATGTCAACCACTTC-3⁣′ Rev: 5⁣′-CAGCGRGTATTTCCGCGTGAAG-3⁣′	NA
*Apoptosis genes*			
Cleaved caspase 3	170	Fwd: 5⁣′-AAGCGAATCAATGGACTCTGG-3⁣′ Rev: 5⁣′-GAATGTTTCCCTGAGGTTTGC-3⁣′	[29]
Bax	195	Fwd: 5⁣′-TGGCAGCTGACATGTTTTCTGAC-3⁣′ Rev: 5⁣′-TCACCCAACCACCCTGGTCTT-3⁣′	[30]
Cytochrome C	132	Fwd: 5⁣′-GGGCGAGAGCTATGTAATGCAAG-3⁣′ Rev: 5⁣′-TACAGCCAAAGCAGCAGCTCA-3⁣′	[31]
*Autophagy genes*			
Beclin 1	191	Fwd: 5⁣′-CAAGATCCTGGACCGTGTCA-3⁣′ Rev: 5⁣′-TGGCACTTTCTGTGGACATCA-3⁣′	[32]
LC3II	167	Fwd: 5⁣′-GATGTCCGACTTATTCGAGAGC-3⁣′ Rev: 5⁣′-TTGAGCTGTAAGCGCCTTCTA-3⁣′	[33]
*p53 and constitutive genes*			
p53	90	Fwd: 5⁣′-TGCGTGTGGAGTATTTGGATG-3⁣′ Rev: 5⁣′-TGGTACAGTCAGAGCCAACCTC-3⁣′	[34]
GAPDH	161	Fwd: 5⁣′-TTGGCTACAGCAACAGGGTG-3⁣′ Rev: 5⁣′-GGGGAGATTCAGTGTGGTGG-3⁣′	[35]

**Table 2:** Growth and biochemical profiles of producing HEK293 clones.

	**HEK293**	**HEK/27**	**HEK/U**	**HEK/27/U-C1**	**HEK/27/U-C4**	**HEK/27/U-C5**
Passage number	8	8	8	9	7	9
CD_max_ (CV/mL)	2.84 × 10^6^	3.4 × 10^6^	3.72 × 10^6^	2.39 × 10^6^	3.14 × 10^6^	5.72 × 10^5^
% viability	85	88	89	96	95	92
*t*_d_ (h)	29	44	36	34	40	38
*μ* (h^−1^)	0.0233	0.0157	0.020	0.0203	0.017	0.0182
*q*Glc (pmol/CV∗d)	−0.908 ± 0.303	−1.150 ± 0.046	−0.564 ± 0.110	−0.504 ± 0.060	−0.766 ± 0.013	−0.666 ± 0.062
*q*Lac (pmol/CV∗d)	1.187 ± 0.339	2.392 ± 0.007	2.627 ± 0.018	2.612 ± 0.070	1.321 ± 0.051	3.692 ± 0.365
*q*Gln (pmol/CV∗d)	−0.296 ± 0.002	−0.139 ± 0.008	−0.077 ± 0.005	−0.244 ± 0.030	−0.218 ± 0.006	−0.109 ± 0.002
*q*Glu (pmol/CV∗d)	−0.045 ± 0.001	−0.013 ± 0.002	−0.042 ± 0.008	−0.006 ± 0.001	−0.014 ± 0.001	0.140 ± 0.029
*Y*_Lac/Glc_ (qLac/qGlc)	1.33 ± 0.069	2.08 ± 0.089	4.853 ± 0.979	5.269 ± 0.768	1.72 ± 0.037	5.539 ± 0.030

*Note:* Maximum cell density: CD_max_; % of viability: percentage of viability reached at the end of the kinetics; time of duplication: *t*_d_; specific growth rate: *μ*; *q*: specific consumption rate; pmol/CV∗D: picomole/viable cell∗day. Signs (−) indicate metabolite consumption. Signs (+) indicate metabolite production.

Abbreviations: Glc, glucose; Gln, glutamine; Glu, glutamate; Lac, lactate.

**Table 3:** Analysis of different fragments and size of UCOE elements.

**UCOE subfragment**	**Cell line**	**Model protein**	**Level of production**	**Reference**
8 Kb	CHO-K1	IgG	↓	[45]
4 Kb	CHO-S	TRAIL R2	↑	[46]
4 Kb	CHO-S	h-TG2	↑	[46]
4 Kb	Mouse embryonic stem cells	GFP	↓	[47]
4 Kb	HEK293	hIFN*γ*	↓	“Present work”
3.2 Kb	CHO-K1	IgG	↑	[45]
3.2 Kb	CHO-K1	IgG	↑	[45]
3.2 Kb	BHK21	Factor VIII	↓	[48]
1.5 Kb	CHO-DG44	TNF*α*	↑	[8]
1.5 Kb	BHK21	Factor VIII	↑	[48]
1.5 Kb	Mouse embryonic stem cells	GFP	↑	[47]
0.7 Kb	Mouse embryonic stem cells	GFP	↑↑	[47]
0.7 Kb	HEK293F	GFP	↑	[43]

*Note:* Production levels were determined with respect to the control clone used in each article.

Abbreviations: GFP, green fluorescent protein; hIFN*γ*, human interferon gamma; h-TG2, human transglutaminase 2; IgG, immunoglobulin G; TNF*α*, tumor necrosis factor *α*; TRAIL R2, human tumor necrosis factor-related apoptosis-inducing ligand R2.

## Data Availability

Data will be made available upon request to the corresponding author.

## Supporting Information

Supporting Information Additional supporting information can be found online in the Supporting Information section. The supporting information contains the sequencing alignments of the UCOE/IFN*γ* and TOPO/HSP27 vectors. These were performed using the BLAST program from NCBI. Figure S1. The alignment of IFN*γ* contained in the UCOE/IFN*γ* vector with the data recorded in the NCBI database is shown. It is observed that there is 100% identity with the coding region of the human IFN*γ*. Figure S2. The alignment of HSP27 (HSPB1) contained in the TOPO/HSP27 vector with the data recorded in the NCBI database is shown. It is observed that there is 100% identity with the coding region of human HSP27.
