# Supplementary material for: Expression of UCOE and HSP27 Molecular Elements to Improve the Stable Protein Production on HEK293 Cells
Source: Biomed Res Int. 2025 Feb 25;2025:5556353. doi: 10.1155/bmri/5556353 (PMC11879590; doi:10.1155/bmri/5556353)
Supplement: Supporting Information — Additional supporting information can be found online in the Supporting Information section. The supporting information contains the sequencing alignments of the UCOE/IFNγ and TOPO/HSP27 vectors. These were performed using the BLAST program from NCBI. Figure S1. The alignment of IFNγ contained in the UCOE/IFNγ vector with the data recorded in the NCBI database is shown. It is observed that there is 100% identity with the coding region of the human IFNγ. Figure S2. The alignment of HSP27 (HSPB1) contained in the TOPO/HSP27 vector with the data recorded in the NCBI database is shown. It is observed that there is 100% identity with the coding region of human HSP27. [file 5556353.f1.docx]

**Supplementary data**

The supplementary material contains the sequencing alignments of the UCOE/IFNγ and TOPO/HSP27 vectors. These were performed using the BLAST program from NCBI.


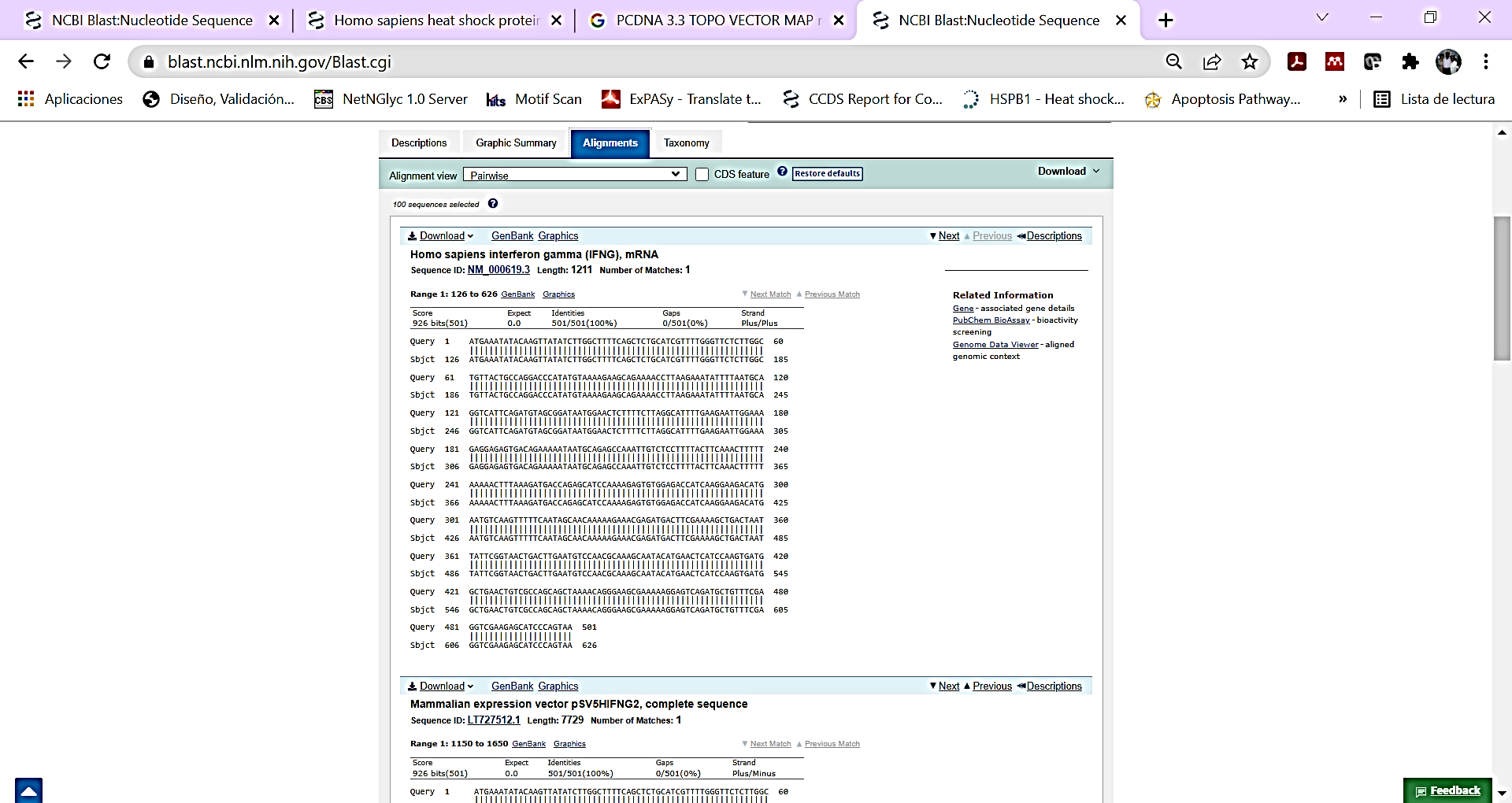


S1. The alignment of the IFNγ contained in the UCOE/IFNγ vector with the data recorded in the NCBI database is shown. It is observed that there is 100% identity with the coding region of the human IFNγ.


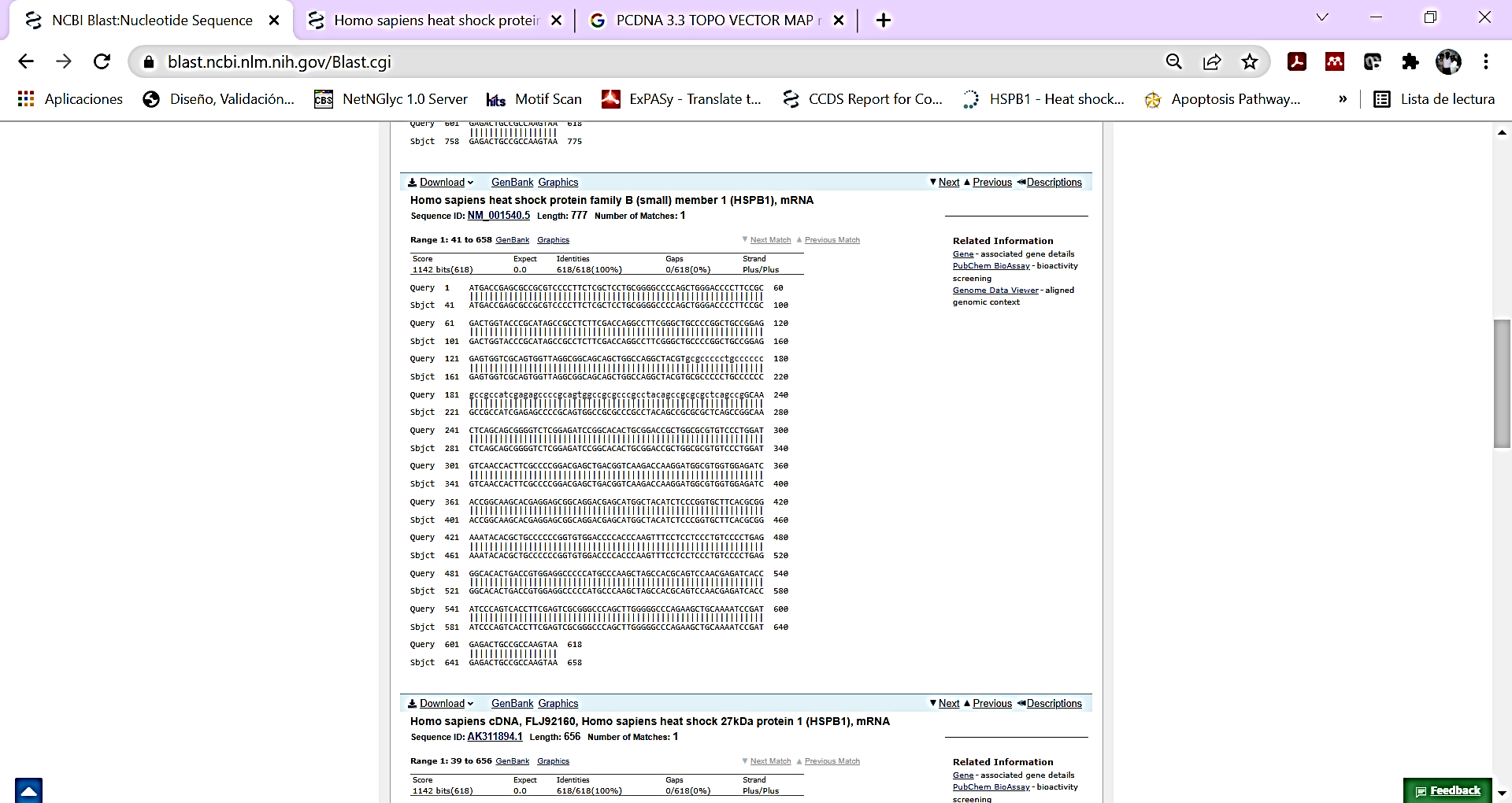


S2. The alignment of HSP27(HSPB1) contained in the TOPO/HSP27 vector with the data recorded in the NCBI database is shown. It is observed that there is 100% identity with the coding region of human HSP27.
